# Homologs of bacterial heat-labile enterotoxin subunit A contribute to development, stress response, and virulence in filamentous entomopathogenic fungus *Beauveria bassiana*

**DOI:** 10.3389/fimmu.2023.1264560

**Published:** 2023-09-22

**Authors:** Jin-Li Ding, Kang Wei, Ming-Guang Feng, Sheng-Hua Ying

**Affiliations:** Institute of Microbiology, College of Life Sciences, Zhejiang University, Hangzhou, China

**Keywords:** *Beauveria bassiana*, enterotoxin, development, virulence, stress response

## Abstract

**Introduction:**

Enterotoxigenic bacteria commonly excrete heat-labile enterotoxins (LT) as virulence factors that consist of one subunit A (LTA) and five B subunits (LTB). In fungi, there are a large number of genes encoding the homologs of LTA, but their biological roles remain largely unknown.

**Methods:**

In this study, we identified 14 enterotoxin_A domain proteins in filamentous fungus *B. bassiana* in which five proteins were functionally characterized.

**Results:**

Five proteins displayed diverse sub-cellular localizations but perform convergent functions in stress response, development, and virulence. The loss of five *LTA* genes resulted in significant reduction in conidial production, blastospore formation, and the increased sensitivity to oxidative and cell wall –perturbing stresses. The virulence of five disruptants was notably weakened as indicated by topical and intrahemocoel injection assays. Notably, the loss of these five proteins led to the significant changes in the carbohydrate profiles of cellular surface, which induced the enhanced host immune reactions of encapsulation and melanization.

**Discussion:**

Thus, LTA proteins contribute to the fungus–host interaction via maintaining the carbohydrate profiles of cellular surface. This study expands our understanding of the enterotoxin_A domain proteins in fungal physiology and deepens mechanisms involved in the lifestyle of fungal insect pathogens.

## 1 Introduction

Enterotoxigenic bacteria commonly employ heat-labile enterotoxins (LT) as virulence factors that deregulate membrane ion channels and cause diarrhea in hosts (1). In terms of architecture, LT is a heterotrimeric toxin composed of a subunit A (LTA) and five B subunits (LTB). LTA acts as the catalytic subunit of toxin, and LTB is responsible for the receptor-binding activity and entry into the intestinal epitheliums (2, 3). In fungi, there are no genes encoding LTB, but there are a large number of genes for LTA (4). In common with other fungi, LTA genes are prevalent in the genomes of fungal entomopathogens. An evolutionary comparative genomic investigation indicated that positive selection on LTA is specific to the ant-infecting genus *Ophiocordyceps*, which suggests that LTAs acts as important factors in host adaptation of the fungi from this genus (5). Although the LTAs have been implicated in the fungus–host interaction, more investigations are needed to elucidate their roles in the physiological process of entomopathogenic fungi.

*Beauveria bassiana*, a typical filamentous entomopathogenic fungus, has a wide host spectrum and is widely used in biological control of arthropod pests (6, 7). The infection cycle of *B. bassiana* against the hosts consists of multiple stages, including conidial adhesion to the host cuticle, conidial germination into invasive hypha, hyphal penetration through the host cuticle, fungal proliferation within the host hemocoel, and growth on cadaver followed by conidiation (8, 9). Thus, *B. bassiana* serves as a model to explore the mechanism involved in the host–pathogen interaction (10). In the host hemocoel, *B. bassiana* secretes LysM effectors to disrupt insect immune defense and to protect fungal cells from hydrolases (11). Secretive ribotoxin is also utilized by *B. bassiana* as a virulence factor that inhibits insect immunity, induces apoptosis in tissues, and retards the host development (12, 13). More investigations are needed to explore the virulence-related factors in entomopathogenic fungi.

This study aimed to decipher the roles of *LTA* genes in vegetative growth, development, and pathogenesis of *B. bassiana*. Here, we identified 14 genes coding LTA, and five of them were functionally characterized. Five LTAs display significant diversities in sub-cellular localizations and physiological phenotypes. Our results highlight the direct genetic evidences for LTs in the fungus–host interaction.

## 2 Materials and methods

### 2.1 Strains, media, and growth conditions

The wild type (WT) of *B. bassiana* ARSEF2860 (Bb2860) was obtained from the U.S. Department of Agriculture Entomopathogenic Fungus Collection (Ithaca, NY, USA) (14). Sabouraud dextrose agar with yeast extract (SDAY) (4% glucose, 1% peptone, and 1.5% agar plus 1% yeast extract) was used to routinely maintain fungal strains at 25°C. *Escherichia coli* DH5α (Invitrogen, Waltham, MA, USA) was cultured in a Luria-Bertani medium with necessary antibiotics for plasmid propagation. Yeast extract broth (YEB) medium (w/v: 0.5% sucrose, 1% peptone, 0.1% yeast extract, and 0.05% MgSO_4_) was used to culture *Agrobacterium tumefaciens* AGL-1, and the resultant bacteria were used as donor cells in fungal transformation. Czapek-Dox agar (CzA) (3% glucose, 0.3% NaNO_3_, 0.1% K_2_HPO_4_, 0.05% KCl, 0.05% MgSO_4_, and 0.001% FeSO_4_ plus 1.5% agar) was used as the chemically synthetic medium in screening transformants and phenotypic assays.

### 2.2 Bioinformatic analysis of enterotoxin_ A domain proteins in *B. bassiana*

Domain annotation was conducted on the predicted proteome of *B. bassiana* (14) through the online portal SMART (http://smart.embl-heidelberg.de). All resultant proteins were named as *B. bassiana* LTA, and their similarities with *Escherichia coli* LTAs were performed with multiple- alignment analysis using MEGA version 7 as described previously (15).

### 2.3 Sub-cellular localization analyses of *B. bassiana* LTA

Plasmid construction was performed as described previously (16). Briefly, the coding sequence of each gene was amplified with the respective primers (PxGFPF/PxGFPR) (X: BbLTAX) using Complementary DNA (cDNA) as the template. The resultant DNA fragments were fused to the 5′-terminus of green fluorescence protein gene in the vector pBbTEF-MCS-Gfp-sur (pBMGS) (16). The resultant p0380-X-gfp-sur was integrated into the WT strain via *Agrobacterium*-mediated transformation. Putative transformants were screened on the selection plates with chlorimuron ethyl (10 μg/mL) and confirmed under a fluorescence microscope. The conidia of transformants were inoculated in SDB (SDAY without agar) and incubated at 25°C for 2 days. Hyphal samples were collected from each culture and stained with 7-amino-4-chloromethylcoumarin (CMAC). Fluorescent signals were visualized under a laser scanning confocal microscope (LSCM).

### 2.4 Targeted gene disruption and complementation

Disruption mutants were generated using a method of homologous replacement coupled with a fluorescence reporter (17). The primer pairs PxUF/PxUR and PxDF/PxDR were used to amplify 5′- and 3′-fragments of the indicated gene, respectively. The resulting fragments were sub-cloned into the restriction enzyme sites (*Xma*I/*Bam*HI and *Xba*I/*Hpa*I) in p0380-GTB using the ClonExpress II One Step Cloning Kit (Vazyme Biotech, Nanjing, China), generating gene disruption vector. The full-length gene together with promoter region was amplified for each gene with primer pair PxHF/PxHR, and the obtained fragment was ligated into the plasmid p0380-sur, generating the complementation vector. The resulting vector was transformed into the gene disruption mutant with the *Agrobacterium*-based transformation method. Putative gene disruption and complementation strains were grown on plates included with phosphinothricin (200 μg/mL) and chlorimuron ethyl (10 μg/mL), respectively, and further identified via PCR analyses with primer pair PxJF/PxJR. The candidate strains were confirmed for their fluorescent signals under a LSCM. All primers are included in **Supplementary Table 1**.

### 2.5 Phenotypic assays

Fungal phenotypes, including vegetative growth, stress response, development, and virulence, were evaluated among the WT, gene disruption, and complemented mutant strains as described previously (18). Fungal strains were cultured on SDAY plates for 7 days at 25°C, and conidia were used as initial inocula in following assays. All experiments were repeated three times.

Vegetative growth: Conidial suspension (1 µL, 10^6^ conidia/mL) was inoculated on the CzA plates modified with various carbon and nitrogen sources. Colony diameter was examined at 7 days after incubation at 25°C. Carbon sources (final concentration, w/v) included glucose (3%), sucrose (3%), fructose (3%), trehalose (3%), olive oil (0.5%) linoleic acid (0.01%), and oleic acid (0.2%). Nitrogen sources (final concentration, w/v) included NH_4_NO_3_ (0.5%) and urea (0.5%).

Stress responses: Fungal responses to oxidative stress were determined on a CzA plate supplemented with 1 mM H_2_O_2_, 2 mM H_2_O_2_, Congo red (3 μg/mL), and Calcofluor white (CFW; 1 μg/mL). A droplet (1 µL) of conidial suspension (10^6^ conidia/mL) was placed on the plate and incubated at 25°C. The colony diameter was measured at 7 days after incubation. CzA plates without stress chemicals were used as control.

Fungal development: Conidiation capacity was determined on SDAY plates. Aliquots (100 μL of 10^7^ conidia/mL) were inoculated on SDAY plates and grown for 7 days at 25°C. Conidia in mycelial discs (5 mm in diameter) were rinsed into 0.02% Tween 80 solution. Cell concentration was quantified and used to calculate conidial yield as conidial number per square centimeter. In addition, the mycelia of the WT and gene disruption mutants were sampled at 4 days after incubation, respectively. The conidium-producing structures were stained with CFW and examined under a LSCM. Fungal development under submerged conditions was evaluated in SDB medium (SDAY without agar). Conidia were inoculated into SDB at the final concentration of 10^5^ conidia/mL and incubated for 3 days at 25°C with shaking. The blastospore concentration was determined, and blastospore yield was displayed as the blastospore number per milliliter of broth.

### 2.6 Interaction between fungus and host

To examine fungal virulence, the last instar *Galleria mellonella* larvae were used as the bioassay insects, and each treatment involved 30–35 larvae. Conidial concentrations in suspension were adjusted to concentrations of 10^5^ and 10^7^ spore/mL, which were used in the intra-hemocoel injection and cuticle infection methods, respectively. For injection assay, a conidial suspension (5 μL, 10^5^ spores/mL) was inoculated into the host hemocoel with a syringe. In cuticle infection assay, the larvae were immersed in suspension for 15 s. All infected insects were reared at 25°C, and the mortality was recorded daily. The median lethal time (LT_50_) was calculated by Kaplan–Meier method with log-rank test for determining the difference between the paired survival trends. In addition, fungal development in the host hemocoel was determined by quantifying the concentration of hyphal bodies in hemolymph at 3 days after infection.

Hemocyte encapsulation assay was performed as described previously (19). Briefly, aliquots of 5-μL conidial suspension were injected into the host hemocoel, and the infected hosts were reared at 25°C. The hemolymph was sampled everyday within 2 days after injection and then every 6 h after 2 days. Fungal cell interactions with host blood cells were recorded under a microscope.

### 2.7 Conidial binding capacity to lectin

Conidial lectin-binding traits were examined as previously described (20). The Alexa Fluor 488–labeled lectins included concanavalin A (ConA), *Galanthus nivalis* lectin (GNL), peanut agglutinin (PNA), and wheat germ agglutinin (WGA) (Vector Laboratories Inc., California, USA). Briefly, conidia were suspended in 0.02% Tween 80 and fixed in 3% volume per volume (*v/v*) formaldehyde for 30 min. The resultant conidia were re-suspended in buffer and labeled with lectin for 1 h in darkness. Unbound lectins were removed by washing. Fluorescent signals were quantified on a CytoFLEX LX flow cytometer (Beckman Coulter Life Sciences, Indianapolis, USA) at the excitation/emission wave lengths of 488/530 nm.

### 2.8 Transcriptional responses of host hemocytes

Real-time PCR analysis was used to reveal the responses of host hemocytes and performed as described previously (19). Conidia (500 cells) of the gene disruption mutant strains were injected into the *G. mellonella* larvae and then were reared at 25°C. Three days later, the larvae were bled at the hind legs, and the haemolyph was collected into 0.2 mL of anticoagulant solution [0.14 M NaCl, 0.1 M glucose, 26 mM citric acid, 30 mM trisodium citrate, and 10 mM EDTA (pH 4.6)]. All manipulations, including bleeding, centrifugation, and washing, were performed at 4°C. The hemocytes were separated from hemolymph by centrifuging and used to extract total RNAs using TRIzol^®^ Reagent (Sigma-Aldrich, Missouri, USA). cDNA was prepared from total RNA with a PrimeScript™ RT Reagent Kit (TaKaRa, Dalian, China). Real-time PCR was used to analyze the expression levels of genes involved in insect immune response. The 18S rRNA gene was used as an endogenous reference, and the relative expression levels were calculated by the 2^−ΔΔC_T_^ method.

### 2.9 Statistical analyses

All measurements for the WT, gene disruption, and complementation strains were subjected to Student’s *t*-test, and the significance was confirmed at *p* < 0.05. Statistical analyses were performed with the software of GraphPad Prism 8 (GraphPad Software, Boston, MA, USA).

## 3 Results

### 3.1 Bioinformatic identification and sub-cellular localization of enterotoxin_ A domain–containing proteins

**Figure 1:**
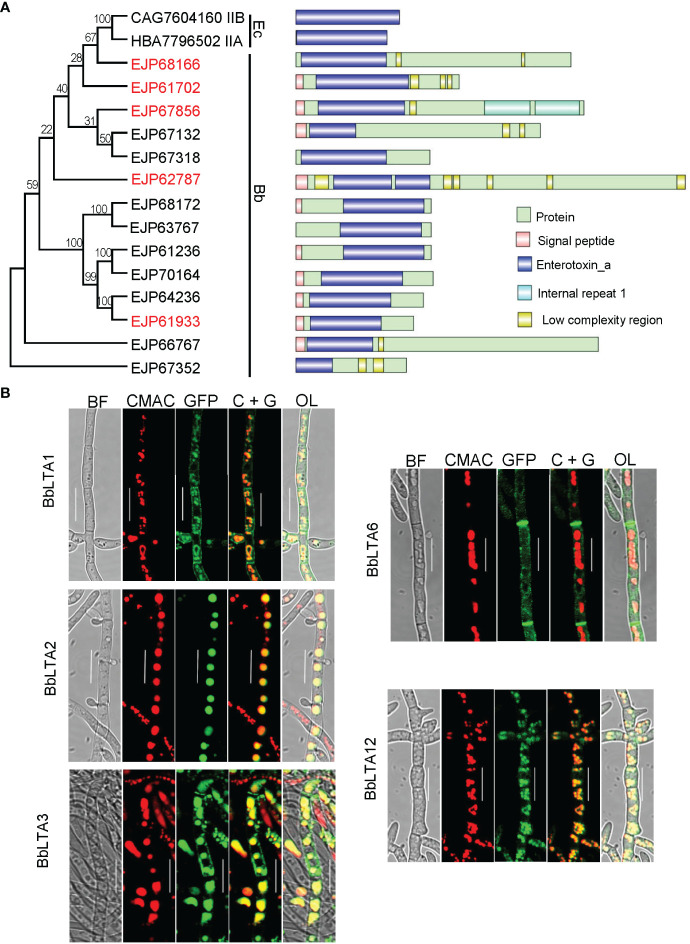
Bioinformatic recognition and sub-cellular imaging of LTA proteins in *B. bassiana*. **(A)** Relationships of bacterial heat-labile enterotoxin subunit A with *B. bassiana* enterotoxin_A–containing proteins (BbLTA). Multiple- sequence alignment was analyzed with neighbor-joining method by 1,000 replicates. GenBank accession number is followed by its gene designation and domain architecture. Domain legends are shown at the right side of the figure. **(B)** Sub-cellular localizations of five LTA proteins. The coding sequence for each protein was fused to GFP gene, and the fusion gene was transformed into the WT strain. The transgenic strain was cultured in SDB medium, and the resultant mycelia were stained with CMAC. Fluorescent signals were examined under a laser scanning confocal microscope. Scale bar, 10 µm.

A domain annotation analysis indicated that there were 14 LTA proteins in *B. bassiana* genome, and each protein contained a domain of enterotoxin_A. The GenBank accession numbers for each protein were EJP68166, EJP61702, EJP67856, EJP67132, EJP67318, EJP62787, EJP68172, EJP63767, EJP61236, EJP70164, EJP64236, EJP61933, EJP66767, and EJP67352. Fourteen proteins were designated successively as BbLTA1 through BbLTA14. The full length and domain size varied among these proteins. In addition, BbLTA1, BbLTA5, BbLTA8, and BbLTA14 did not carry signal peptide, and the other proteins did. As shown in **Figure 1A**, these 14 proteins were grouped into different clusters, and no apparent relationship was noted between domain architecture and phylogeny.

### 3.2 Molecular manipulation of the representative *BbLTA* genes

On the basis of the characteristics of domain organization of BbLTA, five proteins (i.e., BbLTA1, BbLTA2, BbLTA3, BbLTA6, and BbLTA12) were selected as representative ones for functional studies. The N-terminus of the indicated protein was fused with GFP fluorescent protein, and the fluorescent signals indicated the distribution of the fusion proteins. As shown in the **Figure 1B**, the green fluorescence signals of BbLTA1, BbLTA3, and BbLTA12 were distributed on the cell periphery and vacuoles, whereas the green signals of BbLTA2 were exclusively observed in vacuoles and BbLTA6 were localized on hyphal septa. These results indicated that the BbLTA proteins exhibited diverse sub-cellular localizations.

### 3.3 Phenotypic evaluation of fungal development

**Figure 2:**
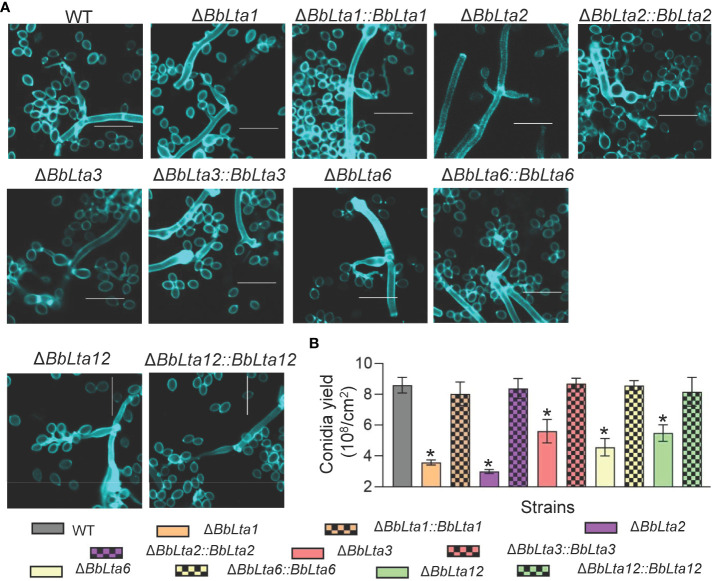
Assays for fungal development into conidia. **(A)** Morphological view of conidium-producing structures. Conidial suspension was inoculated on SDAY plates and cultured for 3 days at 25°C. The sampled mycelia were stained with Calcofluor white and examined under a fluorescence microscope. Scale bars, 10 µm. **(B)** Conidial production. Conidial suspension was smeared on the SDAY plates and cultured for 7 days at 25°C. Conidia on mycelia were quantified, and conidial yield was calculated as spore number per square centimeter. *: *P*<0.05 (Student's *t*-test).

To investigate the roles of BbLTAs, gene disruption and complementation strain were constructed as detailed in Materials and methods section and as shown in **Supplementary Figures 1A, B**. On SDAY plates (aerial condition), the conidia-forming structures of each strain were observed at 3.5 days after incubation. As shown in **Figure 2A**, the WT strain produced normal flask-shaped conidia-forming structures. The disruption mutants of Δ*BbLta2* and Δ*BbLta3* produced the conidia-forming structures with abnormal appearance. No noticeable changes were observed in the sporulating structures of Δ*BbLta1*, Δ*BbLta6*, and Δ*BbLta12* strains, as compared with that of WT. However, these three disruption mutants displayed the significant reduction in conidial production [means ± standard deviation (SD)], when compared with that of the WT strain (**Figure 2A**). The conidial yield of Δ*BbLta1*, Δ*BbLta6*, and Δ*BbLta12* were 3.58 ± 0.14, 5.61 ± 0.62, and 5.48 ± 0.44 (× 10^7^ conidia/cm^2^), respectively, as compared with that of the WT strain (8.59 ± 0.41 × 10^7^ conidia/cm^2^). The decrease degrees for these three mutants were 58.29%, 34.76%, and 36.22%, respectively. Δ*BbLta2* and Δ*BbLta3* produced 3.00 ± 0.11 and 4.56 ± 0.46 (× 10^7^ conidia/cm^2^), respectively. The complementation strains showed no significant difference in production, when compared with that of the WT strain (**Figure 2B**). These results indicated that all gene disruption mutants displayed the significant reduction in conidial production.

**Figure 3:**
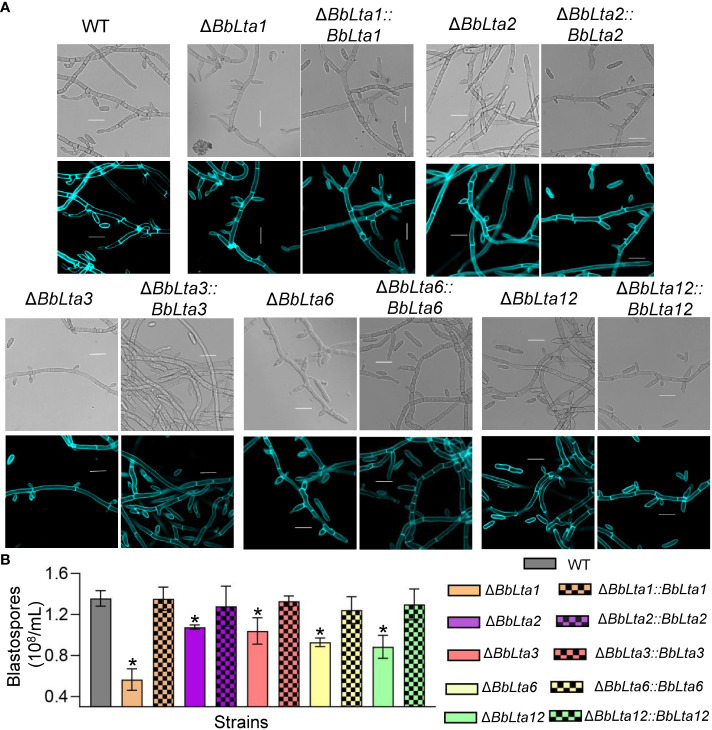
Assays for fungal development under the submerged condition. **(A)** Morphological view of blastospore-producing structures. Conidial suspension was inoculated into SDB medium and cultured for 3 days at 25°C with aeration. The sampled mycelia were stained with Calcofluor white and examined under a fluorescence microscope. Scale bars, 10 µm. **(B)** Blastospore production. Blastospore concentration was quantified and used an index of blastospore yield as blastospore number per milliliter broth. *: *P*<0.05 (Student's *t*-test).

As shown in **Figure 3A**, gene disruption resulted in significant change in formation of the blastospore-producing structures; in particular, Δ*BbLta1* barely formed structures for blastospore generation. The disruption of *BbLTA* genes had significant impacts on the blastospore production (means ± SD) (**Figure 3B**). The blastospore yield of WT strain was 1.36 ± 0.06 × 10^8^ blastospores/mL, and those of Δ*BbLta1*, Δ*BbLta2*, Δ*BbLta3*, Δ*BbLta6*, and Δ*BbLta12* mutant strains were 0.57 ± 0.09, 1.08 ± 0.02, 1.04 ± 0.10, 0.93 ± 0.03, and 0.89 ± 0.09, respectively. The decrease degrees were 58.35%, 20.64%, 23.46%, 31.57%, and 34.77%, respectively. The complementation strains showed no significant difference with the WT strain.

### 3.4 Physiological contributions to lipid utilization

**Figure 4:**
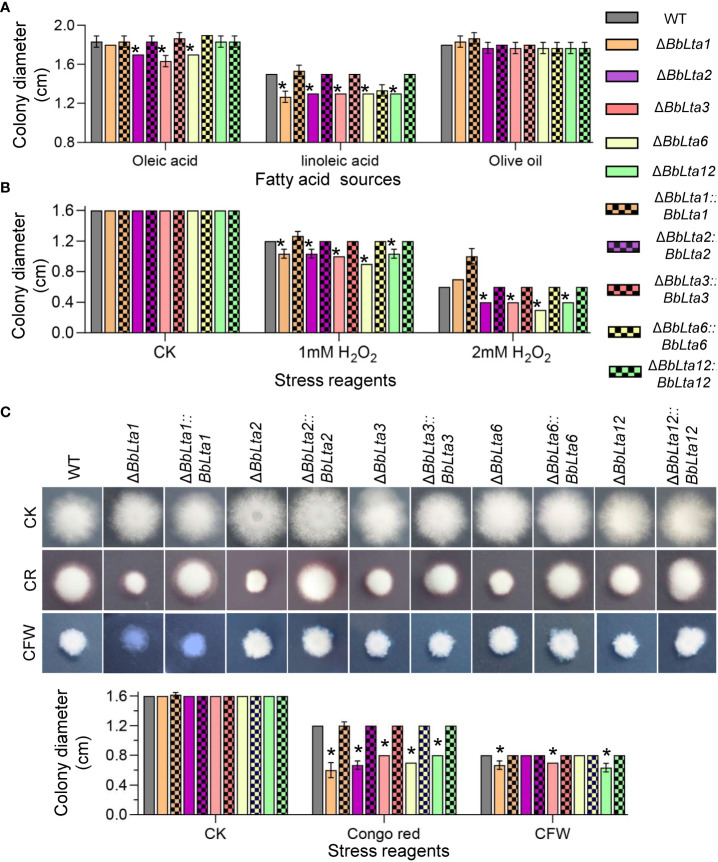
Fungal vegetative growth. **(A)** Fungal growth on lipids. The carbon source in CzA plates was replaced with the indicated lipid. Mycelia growth under oxidative **(B)** and cell wall– perturbing **(C)** stresses were determined on CzA plates included with Congo red (CR) and Calcofluor white (CFW). Conidial suspension (1 µL) was cultured at 25°C, and colony diameter was examined at 7 days after incubation. WT, wild type. Asterisks indicate the significant difference in colony diameter between the wild-type and disruption mutant strains (Student’s *t*-test, * *P* < 0.05).

To determine the *BbLTA* roles in nutrient utilization, the fungal growth was evaluated on different carbon or nitrogen sources. After an incubation of 7 days at 25°C, all disruption mutants displayed the reduced growth on a medium with linoleic acid as carbon source, when compared with the WT strain. On oleic acid plates, the disruption mutants of Δ*BbLta2*, Δ*BbLta3*, and Δ*BbLta6* showed the slight reduction in colony diameter (**Figure 4A**). On media with glucose, sucrose, lactose, trehalose, fructose, and maltose as carbon sources as well as NH_4_Cl, peptone, and gelatin as nitrogen sources, the disruption mutants did not displayed significant change in their colony diameter when compared with the WT (**Supplementary Table 2**).

### 3.5 Fungal responses to oxidative and cell wall– perturbing stress

As shown in **Figure 4B**, on the CzA plates, the colony diameters of all disruption mutants were consistent with that of the WT strain. However, when adding 1 mM H_2_O_2_ into medium, the colony diameters of all mutants were significantly decreased when compared with that of the WT strain. As the H_2_O_2_ concentration increased to 2 mM, the colony diameter of Δ*BbLta1* did not significantly differ with that of the WT strain; however, the other four disruptants still showed the reduced colony diameters when compared with the WT strain.

As shown in **Figure 4C**, on the medium supplemented with Congo red, the colony diameters of Δ*BbLta1*, Δ*BbLta2*, Δ*BbLta3*, Δ*BbLta6*, and Δ*BbLta12* were 0.6 cm, 0.67 cm, 0.8 cm, 0.7 cm, and 0.8 cm, respectively. The decrease degrees were 50.00%, 44.44%, 33.33%, 41.67%, and 33.33%, respectively. In addition, Δ*BbLta1*, Δ*BbLta3*, and Δ*BbLta12* strains exhibited the reduced resistance to cell wall stress induced by CFW.

### 3.6 Fungal *in vivo* development and virulence

**Figure 5:**
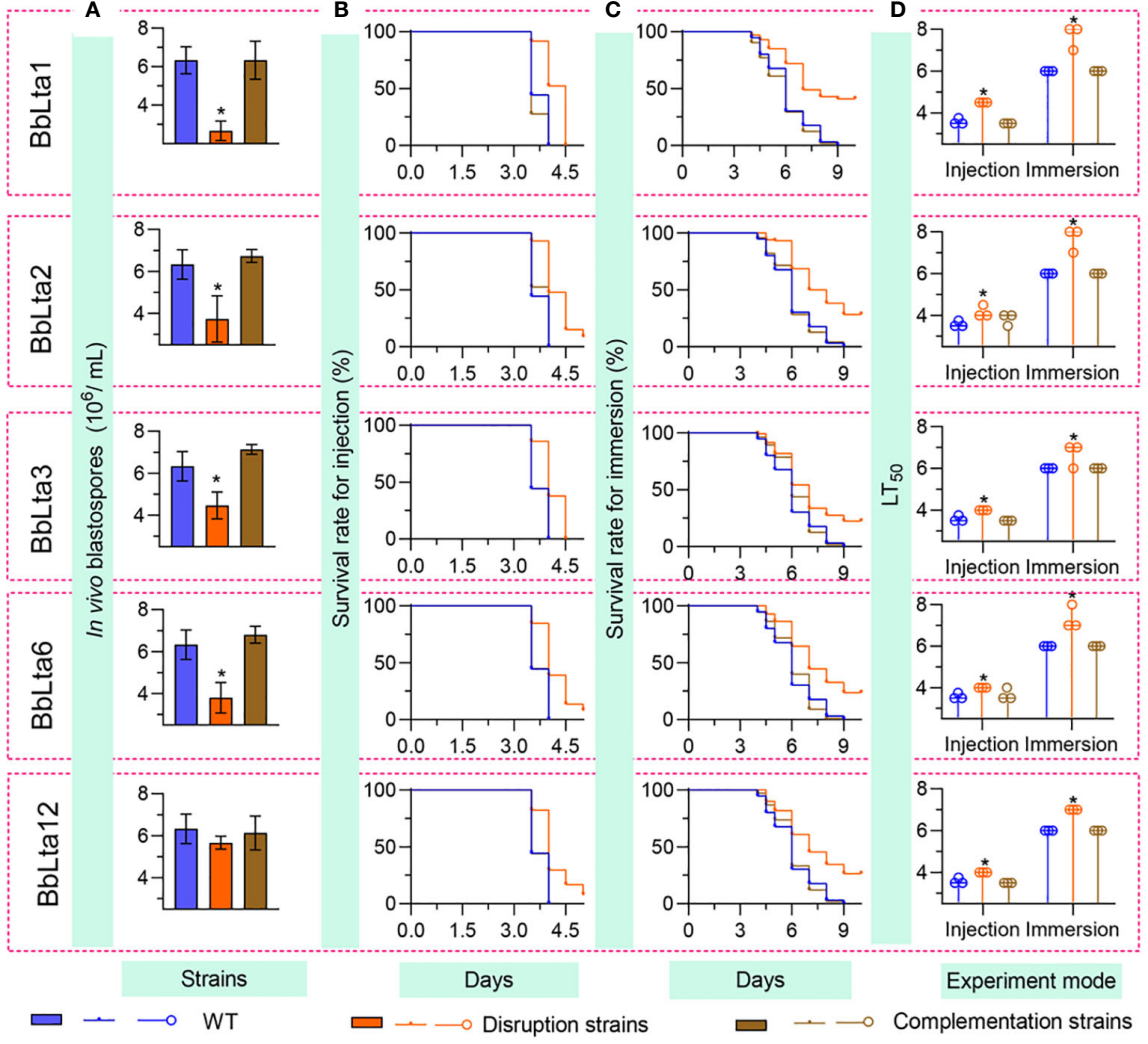
Fungal pathogenic growth and virulence. To evaluate the *in vivo* development, conidia (500 cells) were injected into the host hemoceol, and the infected hosts were cultured at 25°C. **(A)** The *in vivo* blastospore yield was examined at 3.5 days after infection. Fungal virulence was examined by intrahemocoel injection (500 conidia per host) **(B)** and topical inoculation (10^7^ conidia/mL suspension) **(C)**, respectively. The survival percentage was recorded daily and used to calculate the median lethal time (LT_50_) **(D)** using Kaplan–Meier analyses. WT, wild type. * *P* < 0.05 for Student’s *t*-test. Error bars, standard deviation.

Fungal development in the host hemoceol was determined at 3 days after infection. The WT and complemented strains generated plenty of yeast-like hyphal bodies. The *in vivo* blastospore yield (means ± SD) of Δ*BbLta1*, Δ*BbLta2*, Δ*BbLta3*, and Δ*BbLta6* mutant strains was 2.67 ± 0.41, 3.73 ± 0.90, 4.47 ± 0.52, and 3.80 ± 0.59 (× 10^6^ blastospores/mL), respectively (**Figure 5A**). The decrease degrees were 57.89%, 41.05%, 29.47%, and 40.00%, respectively, when compared with that of the WT strain (6.33 ± 0.57 × 10^6^ blastospores/mL). Δ*BbLta12* displayed a slight reduction in blastospore production when compared with that of the WT, but the difference was not statistically significant. No significant difference in blastospore production was observed between the WT and complementation strains. These results indicated that the loss of five BbLTA proteins resulted in the impaired pathogenic growth in the host hemoceol.

Conidial virulence against the greater waxmoth was assessed using two methods: intrahemocoel injection and cuticle inoculation. As shown in **Figures 5B, C**, the survival percentage decreased over time. In both bioassays, the survival curve for each disruptant was significantly lower than that of the WT strain (**Supplementary Table 3**). The LT_50_ (median lethal time) for the WT strain was 3.58 days and 6.00 days in the injection and cuticle inoculation methods, respectively. In the intrahemocoel injection assay, the LT_50_ values for the five disruptants (Δ*BbLta1*, Δ*BbLta2*, Δ*BbLta3*, Δ*BbLta6*, and Δ*BbLta12*) were 4.5 days, 4.2 days, 4.0 days, 4.0 days, and 4.0 days, respectively. In the cuticle inoculation bioassay, the LT_50_ values were delayed by 1.7 days, 1.7 days, 0.7 days, 1.7 days, and 1.0 days, respectively, when compared with that of the WT strain **Figures 5D**. The results indicated that the loss of *BbLTA* genes significantly resulted in the reduced virulence of *B. bassiana*.

### 3.7 Fungal evasion from the host cellular immunity

**Figure 6:**
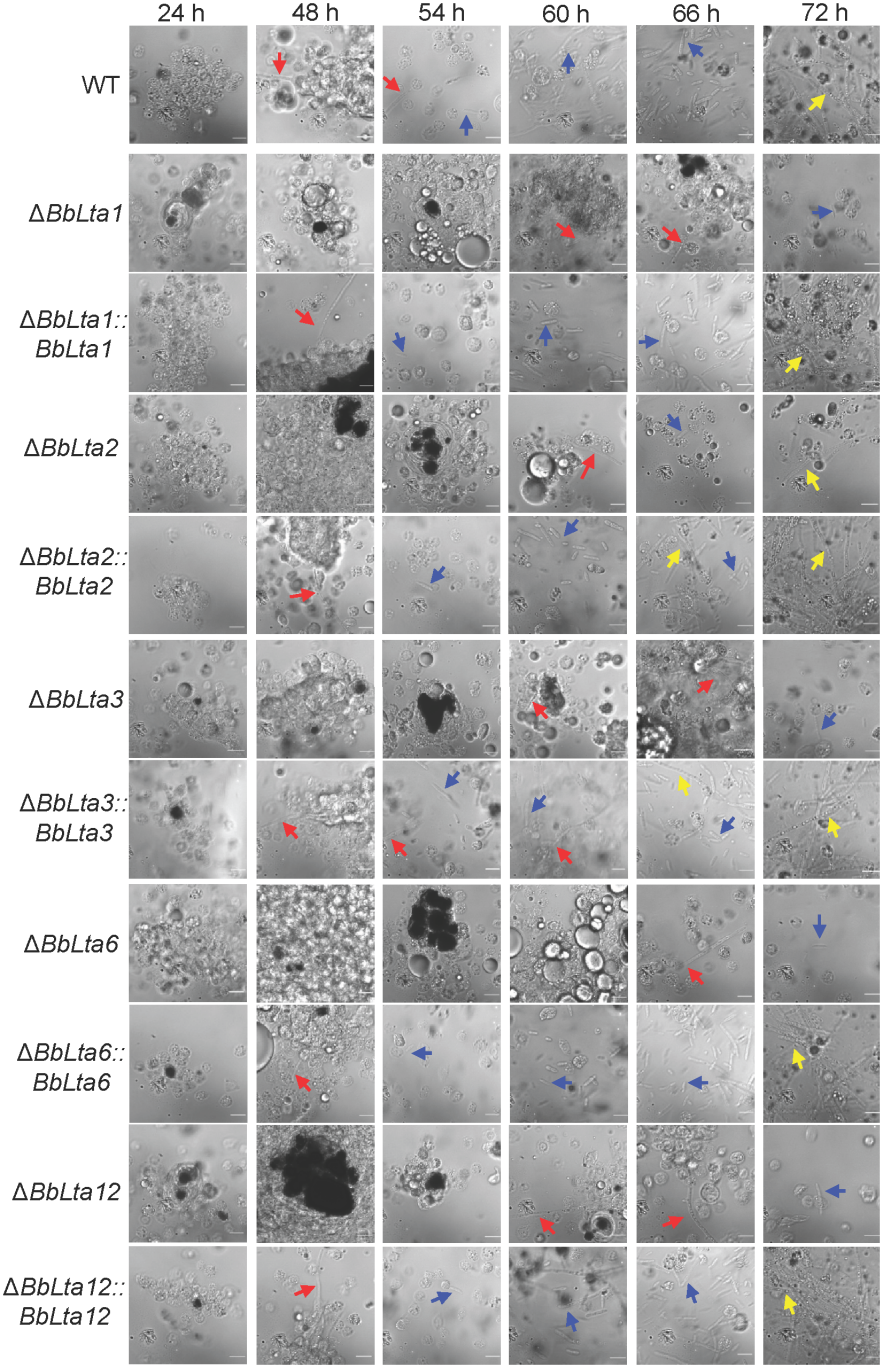
Fungal colonization in the host hemocoel. Conidial concentration (5 µL, 10^5^ spores/mL) was injected into the host hemocoel, and the hosts were incubated at 25°C. The host hemolymph was sampled at 24 h within 2 days and then at the interval of 6 h. Insect hemocyte encapsulation was observed after fungal invasion. After 48 h, the wild-type and complemented strains produced the free-floating hyphal bodies. However, the production of hyphal bodies was significantly delayed in the disruptants, and blastospores were only seen after 60 h. Red and green arrows indicate the fungal cells evading from hemocyte encapsulation and freely floating, respectively. WT, wild type. Scale bars, 10 µm.

Following fungal invasion, host hemocytes congregated and formed melanic dots (21). As shown in **Figure 6**, up to 24 h post-infection (HPI), there were no significant differences in the hemocyte response between each disruptant and the WT strain. However, at 48 HPI, the WT and complemented strains emerged from a cluster of hemocytes and started to produce hyphal bodies, whereas the gene disruption mutants evaded from the host hemocytes until 60 HPI. By 54 HPI, the WT and complemented strains produced numerous free-floating hyphal bodies, whereas the gene disruption mutants produced hyphal bodies until 66 HPI. Nonetheless, the hyphal bodies of the WT and complemented strains underwent differentiation into hyphae at the same time point.

### 3.8 Conidial lectin-binding activity and the host hemocyte response

**Figure 7:**
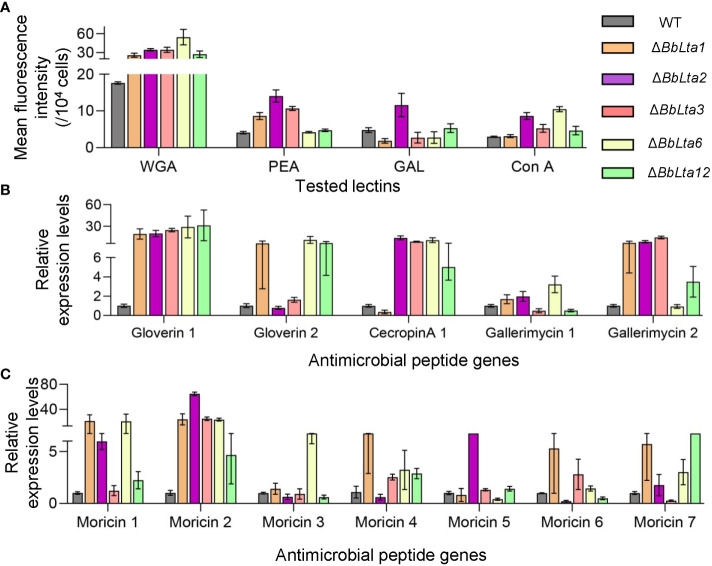
Immune interaction. **(A)** Conidial lectin-binding traits. Lectins included wheat germ agglutinin (WGA), peanut agglutinin (PNA), *Galanthus nivalis* lectin (GNL), and concanavalin A (ConA). Flow cytometry was used examined the bound lectins labeled with fluorescent dye. **(B, C)** Transcriptional analyses of the hemocyte responses. The bioassay insects were challenged by the wild-type (WT) and disruptants, and total RNA was extracted at 3.5 days after infection. qRT-PCR was used to analyze the expression of antimicrobial peptide genes in the host hemocytes.

Flow cytometry analyses revealed that the disruption of *BbLTA* genes altered the lectin-binding traits of conidia (19). Specifically, Δ*BbLta1*, Δ*BbLta2*, Δ*BbLta3*, Δ*BbLta6*, and Δ*BbLta12* mutant strains exhibited a significantly increased ability to bind WGA, whereas the Δ*BbLta1*, Δ*BbLta2*, and Δ*BbLta3* mutant strains showed an enhanced binding ability to PNA. In addition, Δ*BbLta2* displayed an increased ability to bind GAL, and the Δ*BbLta2*, Δ*BbLta3*, Δ*BbLta6*, and Δ*BbLta12* mutant strains showed an enhanced binding ability to ConA (**Figure 7A**).

Moreover, RT-qPCR analyses unraveled that the transcriptional responses of hemocytes challenged by fungal cells. At 3 days after infection, the expression of 12 antimicrobial peptide genes was significantly higher in hemocytes infected by disruptants when compared with those challenged by the WT strain (**Figures 7B, C**). These results indicated that the loss of *BbLTA* genes resulted in the altered transcriptional responses of host hemocytes.

## 4 Discussion

LT act as virulence factors in enterotoxigenic bacteria (1, 22) and consist of a subunit A (LTA) and five B subunits (LTB) (2, 23, 24). In fungi, there are no genes encoding LTB, but the genes for LTA are prevalent (4, 25). In this study, we first characterized the biological roles of the enterotoxin_A domain in the filamentous fungi, using insect pathogenic fungus *B. bassiana* as a model.

In *B. bassiana*, there are 14 enterotoxin_A domain-containing proteins (BbLTA1– BbLTA14). In this study, five representative proteins were functionally characterized. These five proteins display diverse sub-cellular localizations, including cell periphery, vacuole, and hyphal septum, which is owing to diverse domain architectures. In enterotoxigenic bacteria, one A subunit and five B subunits assemble into LT that is excreted via type II secretion route and outer membrane vesicles (2, 26). In bacterial LT, subunit A mediates catalytic reaction. In fungi, there are no genes encoding subunit B (4). These findings suggest that enterotoxin_A domain is evolutionary conserved among microorganisms but functions via different sub-cellular distributions.

Five representative enterotoxin_A domain proteins convergently contribute to fungal development and virulence. Fungal virulence is essential for the biocontrol potential of entomopathogenic fungi in practical application (27, 28). *B. bassiana* penetrates through the host cuticle and invades into the host hemocoel (8). The invasive cells trigger the cellular immune and are wrapped by hemocytes (19). In cellular immune response, the insect hemocytes generate reactive oxygen species (e.g., O^2−^) to inhibit the invasive microbes (29). All tested BbLTA proteins contribute to fungal evasion from the encapsulation by the hemocytes. This result is attributed to, at least, in part, the BbLTA roles in fungal resistance to oxidative stress. In the host hemocoel, *B. bassiana* develops into hyphal bodies via dimorphism mechanism, which is critical for fungal virulence (9, 30). Five BbLTA proteins have convergent roles in fungal development into blastospore, which is determinant for fungal virulence. In *B. bassiana*, other vacuole-related proteins have been verified to be in blastospore formation. BbIMP, a vacuolar membrane protein, is essential for blastospore formation but has a slight role in conidiation (9). A vacuolar protein (VLP4) significantly contributes to conidiation and blastospore formation (31). These findings suggest that the five BbLTA proteins are involved in the sub-cellular architecture of *B. bassiana*, which finally contributes to blastospore development. Insect hemolymph is a hyperosmotic environment for fungal cells (32). Five BbLTA proteins are required for fungal resistance to cell wall–perturbing agents, which implicates their roles in maintaining cell integrity under osmotic stress. These findings suggest the involvements of BbLTA proteins in fungal virulence via assisting cell propagation and resistance to physical and biochemical stresses.

In bacterial interaction with host, LTA is capable of invading cells and elicit the host immune defense responses (26). In *B. bassiana*, five BbLTA proteins contribute to the host–fungus interactions. In humoral immunity, the host produces a plethora of antifungal peptides (AFPs) (e.g., moricin and cecropin) (33, 34). The loss of five BbLTA proteins results in the enhanced expression of AFP genes, which implies that the hosts have the enhanced inhibitory effects on fungal cells without BbLTA proteins. In pathogenic fungi, there are various carbohydrates on cellular surface that mediates pathogen recognition and immune reactions (35, 36). *B. bassiana* cell surface also displayed the stage-specific carbohydrate profiles (20). Five BbLTA proteins contribute to homeostasis of cell wall that is essential for the transcriptional responses of the host hemocytes. In conidia of five *BbLTA*-null strains, the lectin-binding traits have been significantly changed; in particular, the binding activity to WGA has been increased. In *B. bassiana*, a cell wall protein (BbCWP) masks the carbohydrate components in cell wall and prevents cells from immune recognition and activation (19). These findings suggest that BbLTA proteins contribute to the fungus–host interaction via maintaining the homeostasis of carbohydrate profiles on cell surface. On the whole, the enterotoxin_A domain proteins play important roles in the host–pathogen interaction, but the involved mechanisms differ among bacterial and fungal species.

## 5 Conclusion

Together, we identified 14 enterotoxin_A domain proteins in filamentous fungus *B. bassiana* in which five proteins were functionally characterized. Five proteins displayed diverse sub-cellular localizations but perform convergent functions in stress response, development, and virulence. Notably, these proteins contribute to the fungus–host interaction via maintaining the carbohydrate profiles of cellular surface. This study highlights the roles of the enterotoxin_A domain proteins in filamentous fungi and improves our understanding of molecular pathogenesis of fungal insect pathogens.

## Data availability statement

The raw data supporting the conclusions of this article will be made available by the authors, without undue reservation.

## Author contributions

J-LD: Conceptualization, Data curation, Formal Analysis, Funding acquisition, Investigation, Writing – original draft. KW: Formal Analysis, Investigation, Writing – original draft. M-GF: Writing – review & editing. S-HY: Conceptualization, Funding acquisition, Project administration, Supervision, Writing – review & editing.
