# Supplementary material for: Homologs of bacterial heat-labile enterotoxin subunit A contribute to development, stress response, and virulence in filamentous entomopathogenic fungus Beauveria bassiana
Source: Front Immunol. 2023 Sep 22;14:1264560. doi: 10.3389/fimmu.2023.1264560 (PMC10556748; doi:10.3389/fimmu.2023.1264560)
Supplement: Supplementary file 1 [file DataSheet_1.docx]

Supplementary Material

# Supplementary Figures and Tables

## Supplementary Figures


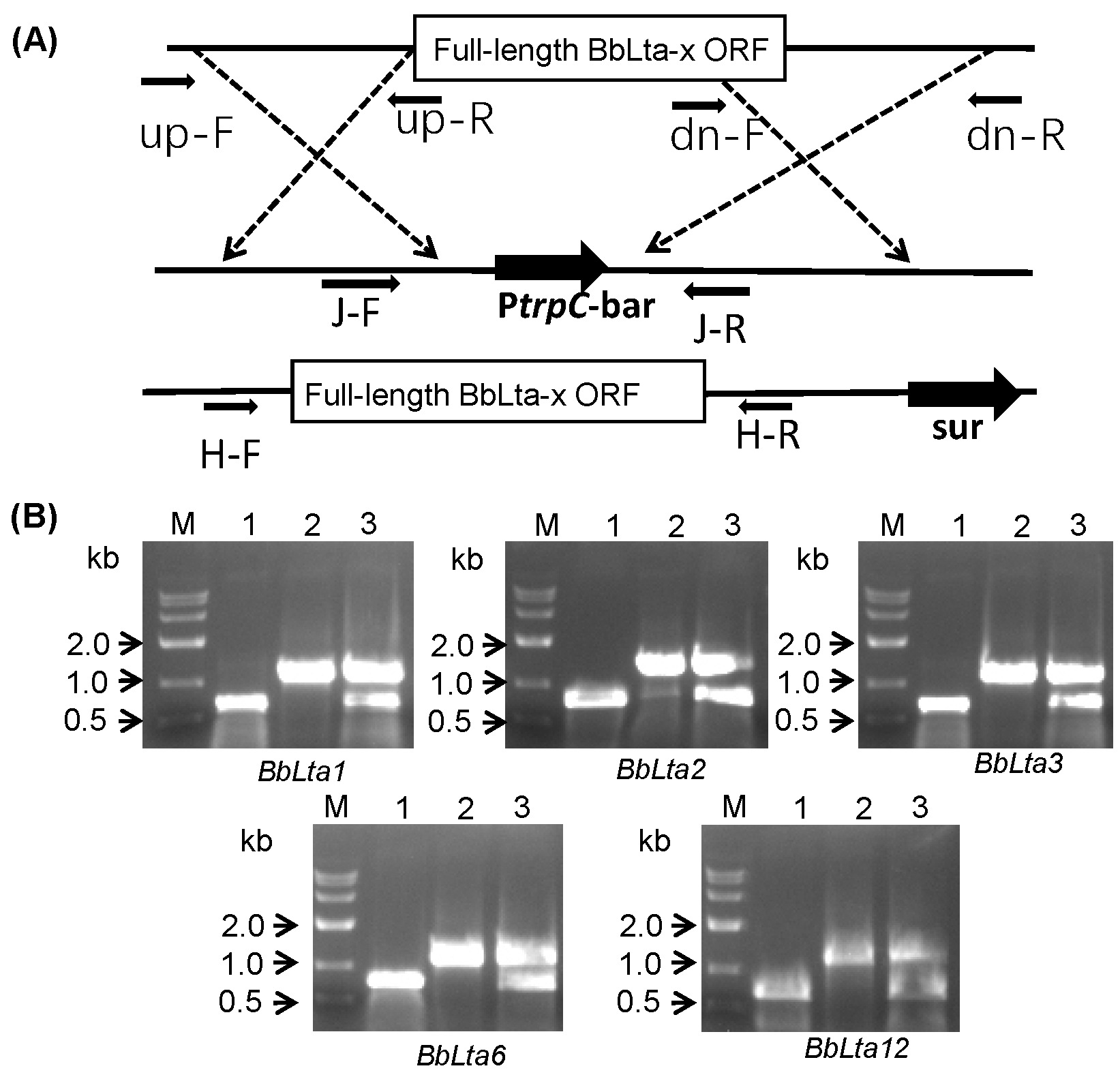


**Supplementary Figure 1.** Gene disruption and complementation in *B. bassiana*. (A) A diagram deciphering primer design for gene disruption and complementation. Gene disruption and complementation were performed via target replacement and ectopic insertion, respectively. BbLTAx represents five representative genes for functional analysis. (B) PCR validation of recombination events. Lane1: wild type, lane 2: disruption mutant, lane 3: reconstituted strain, lane M: DNA marker.

## Supplementary Tables

**Supplementary Table 1** Primers this study.

| **Primers** | **Paired sequences (5′−3′)^*^** | **Purpose** |
| --- | --- | --- |
| PLta1GFPF/ PLta1GFPR | TTCAATCACAAACACCTTCAAAATGAAGCATCTAAAGATTCTATTG/CTCCTCGCCCTTGCTCACCATTTTAAGCTCTATCCAATCGCTT | Amplifying BbLta1  cDNA |
| PLta1UF/ PLta1UR | CGAGCTGTACAAGTAACCCGGGTGAAACATGCCTAATTCTTG/TTGGCTGCAGGTCGACGGATCCTTCAAACTTGCCTTTGGT | Amplifying 5′-fragment of  BbLta1 |
| PLta1DF/ PLta1DR | CGACCCATGGCTCGAGTCTAGAAGAGACTCAAATAGAGCCAAGC/GGTGGTGGTGGCTAGCGTTAACAAAGTTGACTGCCGATGC | Amplifying 3′-fragment of  BbLta1 |
| PLta1HF/ PLta1HR | ATCCGTCGACCTGCAGCCAAGCTTTGAAACATGCCTAATTCTTG/ACACTAGTCAGATCTTCTAGTGTAAAGTTGACTGCCGATGC | Cloning the BbLta1 full  ORF for gene  complementation |
| PLta1JF/ PLta1JR | GTCGTTACAGGCTGGTCC/GCAGTGTATCATTCGGTCT | Screening transformants |
| PLta2GFPF/ PLta2GFPR | TTCAATCACAAACACCTTCAAAATGAAGCTGCTTTGGCTGTTTCTG/CTCCTCGCCCTTGCTCACCATTAGCTTGCTATGACCCCCACGCC | Amplifying BbLta2  cDNA |
| PLta2UF/ PLta2UR | CGAGCTGTACAAGTAACCCGGGACAACATCAAGAAGCCTCAC/TTGGCTGCAGGTCGACGGATCCTTATCTGGCGGGATGTAGTT | Amplifying 5′-fragment of  BbLta2 |
| PLta2DF/ PLta2DR | CGACCCATGGCTCGAGTCTAGACCCCTATACAGACATCATTT/GGTGGTGGTGGCTAGCGTTAACTCCTTGGTCTGTCTATCCTC | Amplifying 3′-fragment of  BbLta2 |
| PLta2HF/ PLta2HR | ATCCGTCGACCTGCAGCCAAGCTTGCTATCGGGAACAGGGTC/ACACTAGTCAGATCTTCTAGTGTAAATGGCTGAGCGTGTAT | Cloning the BbLta2 full  ORF for gene  complementation |
| PLta2JF/ PLta2JR | ACACGAGGCAAGCAAGTA/ATCCAAGACTTCGCTAAACT | Screening transformants |
| PLta3GFPF/ PLta3GFPR | TTCAATCACAAACACCTTCAAAATGCAAGGTCAACACTACTTGTCGC/CTCCTCGCCCTTGCTCACCATGCACCCCTGAGGCTGGTCTACT | Amplifying BbLta3  cDNA |
| PLta3UF/ PLta3UR | CGAGCTGTACAAGTAACCCGGGTCCTTATCGTCGTAATCTGA/TTGGCTGCAGGTCGACGGATCCCTCTGAATGCCAAGGTTT | Amplifying 5′-fragment of  BbLta3 |
| PLta3DF/ PLta3DR | CGACCCATGGCTCGAGTCTAGATCCAGCCCAACAAGAACA/GGTGGTGGTGGCTAGCGTTAACGTCGCACTGCTCATCACG | Amplifying 3′-fragment of  BbLta3 |
| PLta3HF/ PLta3HR | ATCCGTCGACCTGCAGCCAAGCTTTGGCACCGACTACTTGAA/ACACTAGTCAGATCTTCTAGTGTGACCAGCAAACCAAGGAG | Cloning the BbLta3 full  ORF for gene  complementation |
| PLta3JF/ PLta3JR | CCAGCCCGCGAACAGTAT/GCACCCAGGATGCGATTT | Screening transformants |
| PLta6GFPF/ PLta6GFPR | TTCAATCACAAACACCTTCAAAATGAGAGTCTTTACCGCTCGACAGTC/CTCCTCGCCCTTGCTCACCATAGCAGCCGGAGCAGCCTGACCTTCC | Amplifying BbLta6  cDNA |
| PLta6UF/ PLta6UR | CGAGCTGTACAAGTAACCCGGGTTCTACTTGGATGAGGCTCG /TTGGCTGCAGGTCGACGGATCCGAACGACCAGAGGCAACG | Amplifying 5′-fragment of  BbLta6 |
| PLta6DF/ PLta6DR | CGACCCATGGCTCGAGTCTAGAAGGAGGCAGAGGCAGGCAGC /GGTGGTGGTGGCTAGCGTTAACGGCAACCTTGGACGGAATCT | Amplifying 3′-fragment of  BbLta6 |
| PLta6HF/ PLta6HR | ATCCGTCGACCTGCAGCCAAGCTTAGATTGTTTATTTGGTGGGTG/ACACTAGTCAGATCTTCTAGTGTCTGTGGTGCTGTAAGGGTTC | Cloning the BbLta6 full  ORF for gene  complementation |
| PLta6JF/ PLta6JR | CGGTAGAGTAGCGTAGTCGG/CCGCCATTCTTCTCCATAAA | Screening transformants |
| PLta12GFPF/ PLta12GFPR | TTCAATCACAAACACCTTCAAAATGATTAATCCAATGAATTGGGCT/CTCCTCGCCCTTGCTCACCATGACCACGAGCCACTTGGCATTCAT | Amplifying BbLta12  cDNA |
| PLta12UF/ PLta12UR | CGAGCTGTACAAGTAACCCGGGTCGCAACTATACACCCAAAT/TTGGCTGCAGGTCGACGGATCCATGTAAATGTCGGTTCTGGT | Amplifying 5′-fragment of  BbLta12 |
| PLta12DF/ PLta12DR | CGACCCATGGCTCGAGTCTAGAGCGTCCGCTACGACCAGAT/GGTGGTGGTGGCTAGCGTTAACTGCCGCTGGGTAAATCTG | Amplifying 3′-fragment of  BbLta12 |
| PLta12HF/ PLta12HR | ATCCGTCGACCTGCAGCCAAGCTTCTGAAACGCCCTCACATA/ACACTAGTCAGATCTTCTAGTGTAAAGCCATCCGACAGACA | Cloning the BbLta12 full  ORF for gene  complementation |
| PLta12JF/ PLta12JR | CGGTAGAGTAGCGTAGTCGG/CCGCCATTCTTCTCCATAAA | Screening transformants |
| Gloverin-1F/ Gloverin-1R | TATTGGACCAGTCTAATC/ATATTCAATGACAACAGAG | qPCR analyses for Gloverin-1 gene expression |
| Gloverin-2F/ Gloverin-2R | TAATAAGAATGGCGATGT/ATGAAGTTGTGCTGATAT | qPCR analyses for Gloverin-2 gene expression |
| Cecropin A-1F /Cecropin A-1R | ATATTCCTGTTCGTGTTC/CAGCCTTAATGATACCAT | qPCR analyses for Cecropin A gene expression |
| Defensin-1F/ Defensin-1R | GTTGGTCTGCCTATCATT/GCAATCAGAAGTGTAATTCG | qPCR analyses for Defensin-1 gene expression |
| Defensin-2F/ Defensin-2R | AACTGTTTTGTTGATGGT/AATCAGAGGTGTAATTCG | qPCR analyses for Defensin-2 gene expression |
| Defensin-3F/ Defensin-3R | AAGATAGAAGTGTGCTGAA/CGGAACACGATTAGGAAT | qPCR analyses for Defensin-3 gene expression |
| Moricin-1F/ Moricin-1R | CTCTTTATAGGGTCAAATGA/GCTGCACTGATTACTTTA | qPCR analyses for Moricin-1 gene expression |
| Moricin-2F/ Moricin-2R | ATGATGGTGATGGCTATG/TATTGATTCCACGCAGAG | qPCR analyses for Moricin-2 gene expression |
| Moricin-3F/ Moricin-3R | TGCTCGCCCTGTTTGTTG/CACCAATTACACCAAGACCTTT | qPCR analyses for Moricin-3 gene expression |
| Moricin-4F/ Moricin-4R | TCTATTCCTCATGTTCAT/AGACCCTTTTTAATAATTTTG | qPCR analyses for Moricin-4 gene expression |
| Moricin-5F/ Moricin-5R | CTCTTTCTCATGATTATGG/ATAATTTTGCCACCTTTC | qPCR analyses for Moricin-5 gene expression |
| Moricin-6F/ Moricin-6R | CGGTTTATTCCTTATGATTATG/TATAGCTTTACCACCCTTT | qPCR analyses for Moricin-6 gene expression |
| Moricin-7F/ Moricin-7R | AGTTCTTCAATCTCGTAT/AATAATTTTTCCGCCTTT | qPCR analyses for Moricin-7 gene expression |
| GM 18SF/ GM 18SR | AACCTGTTAAGAGACTGTAT/GCCTGTGTAGTGAGTAAT | qPCR analyses for 18sRNA |

*: The underlined region is required for homologous recombination during plasmid construction.

**Supplementary Table 2** Colony diameter of the wild-type (WT), disruption mutant and complementation mutant strains.

| **Strains** | **Carbon and nitrogen sources (Mean)** | | | | | | | | |
| --- | --- | --- | --- | --- | --- | --- | --- | --- | --- |
|  | Glucose | Sucrose | Lactose | Trehalose | Fructose | Maltose | NH_4_Cl | Peptone | Gelatin |
| WT | 1.60 | 1.7 | 1.3 | 1.7 | 1.2 | 1.5 | 2 | 2.8 | 2.7 |
| Δ*Bblta1* | 1.60 | 1.7 | 1.3 | 1.7 | 1.2 | 1.5 | 2 | 2.8 | 2.7 |
| Δ*Bblta1:: Bblta1* | 1.60 | 1.7 | 1.3 | 1.7 | 1.2 | 1.5 | 2 | 2.8 | 2.7 |
| Δ*Bblta2* | 1.60 | 1.7 | 1.3 | 1.7 | 1.2 | 1.5 | 2 | 2.8 | 2.7 |
| Δ*Bblta2:: Bblta2* | 1.60 | 1.7 | 1.3 | 1.7 | 1.2 | 1.5 | 2 | 2.8 | 2.7 |
| Δ*Bblta3* | 1.60 | 1.7 | 1.3 | 1.7 | 1.2 | 1.5 | 2 | 2.8 | 2.7 |
| Δ*Bblta3:: Bblta3* | 1.60 | 1.7 | 1.3 | 1.7 | 1.2 | 1.5 | 2 | 2.8 | 2.7 |
| Δ*Bblta6* | 1.60 | 1.7 | 1.3 | 1.7 | 1.2 | 1.5 | 2 | 2.8 | 2.7 |
| Δ*Bblta6:: Bblta6* | 1.60 | 1.7 | 1.3 | 1.7 | 1.2 | 1.5 | 2 | 2.8 | 2.7 |
| Δ*Bblta12* | 1.60 | 1.7 | 1.3 | 1.7 | 1.2 | 1.5 | 2 | 2.8 | 2.7 |
| Δ*Bblta12:: Bblta12* | 1.60 | 1.7 | 1.3 | 1.7 | 1.2 | 1.5 | 2 | 2.8 | 2.7 |

**Supplementary Table 3** Log-rank tests were performed between the wild-type and individual mutant strains.

| Strains | Injection infection | | Topical infection | |
| --- | --- | --- | --- | --- |
|  | *χ^2^* value | *P* value | *χ^2^* value | *P* value |
| Δ*Bblta1* | 90.87 | <0.0001 | 39.48 | <0.0001 |
| Δ*Bblta2* | 91.80 | <0.0001 | 44.32 | <0.0001 |
| Δ*Bblta3* | 61.67 | <0.0001 | 17.61 | <0.0001 |
| Δ*Bblta6* | 60.94 | <0.0001 | 32.12 | <0.0001 |
| Δ*Bblta12* | 46.83 | <0.0001 | 26.10 | <0.0001 |
